# Optimal Control Based Stiffness Identification of an Ankle-Foot Orthosis Using a Predictive Walking Model

**DOI:** 10.3389/fncom.2017.00023

**Published:** 2017-04-13

**Authors:** Manish Sreenivasa, Matthew Millard, Martin Felis, Katja Mombaur, Sebastian I. Wolf

**Affiliations:** ^1^Optimization in Robotics and Biomechanics, Interdisciplinary Center for Scientific Computing, Heidelberg UniversityHeidelberg, Germany; ^2^Clinic for Orthopedics and Trauma Surgery, Heidelberg University HospitalHeidelberg, Germany

**Keywords:** pathological gait, neuromechanics, movement prediction, model-based optimization, parameter identification

## Abstract

Predicting the movements, ground reaction forces and neuromuscular activity during gait can be a valuable asset to the clinical rehabilitation community, both to understand pathology, as well as to plan effective intervention. In this work we use an optimal control method to generate predictive simulations of pathological gait in the sagittal plane. We construct a patient-specific model corresponding to a 7-year old child with gait abnormalities and identify the optimal spring characteristics of an ankle-foot orthosis that minimizes muscle effort. Our simulations include the computation of foot-ground reaction forces, as well as the neuromuscular dynamics using computationally efficient muscle torque generators and excitation-activation equations. The optimal control problem (OCP) is solved with a direct multiple shooting method. The solution of this problem is physically consistent synthetic neural excitation commands, muscle activations and whole body motion. Our simulations produced similar changes to the gait characteristics as those recorded on the patient. The orthosis-equipped model was able to walk faster with more extended knees. Notably, our approach can be easily tuned to simulate weakened muscles, produces physiologically realistic ground reaction forces and smooth muscle activations and torques, and can be implemented on a standard workstation to produce results within a few hours. These results are an important contribution toward bridging the gap between research methods in computational neuromechanics and day-to-day clinical rehabilitation.

## 1. Introduction

The clinical treatment of neuromuscular gait abnormality is a complex process that demands significant investment of time and effort from the patient (and caregivers), surgeons and orthotists. Often there may be multiple suitable treatment regimes (surgery, orthotics, rehabilitation exercise, etc.) without a clear indication of an optimal choice. The use of computational methods can assist in these decisions in two ways. First, by estimating internal physiological states that cannot be directly measured to help understand the pathology. Second, by predicting the change in such states under manipulation of virtual patient models to help understand the effects of the possible interventions. There is a growing number of studies that apply the former, so called inverse methods, to healthy and pathological movements, e.g., (Nakamura et al., 2005; Damsgaard et al., 2006; Delp et al., 2007; Erdemir et al., 2007; Sreenivasa et al., 2015; Choi et al., 2016). By matching recorded kinematics and ground reaction forces, one may solve for muscle activations under various optimization criteria (Jonkers et al., 2003; Thelen et al., 2003; Erdemir et al., 2007; Groote et al., 2016). Another approach is to use the concept of modularity in neural and muscle recruitment to generate a low dimensional manifold of control signals. Sartori et al. (2013) used this approach to generate EMG signals and joint moments for a lower body neuromuscular model. There are far fewer examples that explore the possibility of predicting the kinematics and dynamics of the body during gait. Here we distinguish between methods that can predict muscle forces *given* body movements, and those that can predict both muscle forces *and* body movements. This work focuses on the latter by applying optimal control based methods to predict movements, ground reaction forces and neuromuscular dynamics during walking with and without an orthosis. The goal here is to support an important clinical routine—fitting of an orthosis to a patient—with the use of computational methods and patient-specific models.

The ideal combination of model/method would be one that is computationally efficient, includes neuromuscular dynamics, produces realistic ground reaction forces, can be tuned to an individual (healthy or pathological) quickly and accurately, and can predict movements. Each of these requirements is challenging, however, methodological and technological advances have made some of these possible. Anderson and Pandy (2001) famously used 10,000 h on a Cray super-computer to solve for a metabolically efficient gait for a lower-body neuromuscular model. More recently Wang et al. (2012) and Dorn et al. (2015) predicted gait patterns for their models with around 1,000 CPU-hours of processing. This level of computational infrastructure and the long time to a solution is not feasible for routine clinical work. In contrast, the works of Schultz and Mombaur (2010), Ren et al. (2007), Felis et al. (2013), Felis and Mombaur (2016), and Srinivasan et al. (2008, 2009) produce results using desktop computers in less than an hour. While the faster solution time is impressive, these works do not include a representation of the muscles, which is necessary to address most clinical questions.

Ackermann and van den Bogert (2010) and Dorn et al. (2015) included muscles and activation dynamics, however, their results were accompanied by ground reaction force peaks that were twice as large as would be expected from healthy human walking. Using an alternative reflex-feedback approach, Geyer and Herr (2010) produced a muscle and reflex-driven simulation of walking that produced ground reaction force profiles that had a comparable form and magnitude to healthy human walking. While these results are impressive, it would be challenging to estimate individualized reflex parameters, especially in a clinical setting.

An alternative to the model-based approaches presented so far is the use of methods from machine learning to adaptively adjust assitive devices to the user. Autonomous learning methods have found application in clinical rehabilitation related to functional electrical stimulation (see e.g., Abbas and Chizeck, 1995; Chang et al., 1997; Ferrante et al., 2004). However, these methods typically require pre-existing datasets and/or a large number of training trials. This makes their extension to the prediction of whole body neuromechanics challenging, as patient data may be sparse or not available at all.

In addition to these computational aspects, a major challenge that must be addressed is the validation of the models and the simulation results. This is a multi-faceted issue that needs to be dealt with at both the technical and clinical fronts. For example, for neuromuscular models a common hurdle is that internal neurological states cannot be measured *in vivo*, and surface EMG can only roughly approximate muscle function (Farina et al., 2014). In addition, a prospective clinical trial is a major undertaking that needs a close collaboration between research and clinical teams. As an initial step, studies such as the one presented here can at the very least compare their results to those measures that are relatively easy to record (e.g., joint kinematics, ground reaction forces, surface EMG). While this is not a full validation, a model that can match these observations can at least assure the clinician of exhibiting behavior that is physiologically realistic.

In the following we detail a patient-specific model and formulate an optimal control problem (OCP) to identify the optimal individualized stiffness of an ankle foot orthosis that minimizes muscle effort while walking. It is important to note that the identification of the stiffness parameters occurs in advance of the patient walking with the orthosis. We do not identify the stiffness parameters from experimental data, but rather predict what the parameters should be for that patient. In general, an OCP defines a minimization problem where an objective function is minimized while abiding the dynamics describing a physical system (in our case the human body + orthosis dynamics). Such methods have been used successfully for robot and human motion generation in the past (Bobrow et al., 1985; von Stryk and Schlemmer, 1994; Schultz and Mombaur, 2010), and to a limited extent for the design of human-assistive devices (Koch and Mombaur, 2015; Mombaur, 2016). In the current work, we strike a balance between model complexity and computational efficiency by modeling the muscles as lumped torque generators rather than anatomically equivalent line-type actuators. The solutions combine physically consistent neuromuscular dynamics and ground-contact dynamics, and can be achieved in a matter of hours on a standard desktop computer. We implement several OCPs that mimic the patient condition as he walked barefoot as well as with an orthosis. Note that in these OCPs we predict movements, joint torques and ground-reaction forces. For the orthosis OCP, we evaluate two cost functions, one that only minimizes muscle effort, and another that minimizes muscle effort while favoring a higher walking speed. In addition we also present a dynamic fit of the model to the recorded gait kinematics. Our simulation results are compared to experimental recordings from a 7-year old patient with neuromuscular deficits.

## 2. Methods

### 2.1. Patient data

Gait data of a 7-year old male (weight 24.7 kg, height 1.25 m) are retrospectively used in this study. The patient presented with multiple bony deformities of neuromuscular origins, which were corrected in a single event multilevel surgery 1.5 years prior to the recording of the gait data. At the time of recordings he presented with a mild crouch, slow walking speed and unstable gait. Recordings were made of the patient walking on level ground with bare feet and with bilateral ankle-foot orthosis. The orthosis stiffness (see Section 2.2.3 for details) was tuned manually by an orthopedic professional overseeing the recordings. Positions of 35 reflective markers attached to the patient's limbs and torso were recorded at 120 Hz during level gait using a 10-camera Vicon system (Vicon, UK). Simultaneous ground reaction forces were recorded at 1080 Hz using Kistler force plates (Kistler GmbH, Germany).

In total 13 barefoot left and right steps and 12 orthosis left and right steps were recorded that contained gait kinematics suitable for further processing. From this set, 3 barefoot left steps and 2 barefoot right steps, as well as 5 orthosis left steps and 4 orthosis right steps, had suitable recorded ground reactions forces. The reduced number of trials with valid ground reaction forces highlight the experimental difficulties associated with getting an under-age patient with neuromuscular deficits to step cleanly on the successive force-plates. The gait recordings were part of a standard clinical routine. Written informed consent was obtained from the parents and the subject. The recordings were conducted according to the guidelines of the Declaration of Helsinki 2013 and approved by the ethics committee of the Medical Faculty Heidelberg of Heidelberg University.

### 2.2. Model formulation

**Figure 1:**
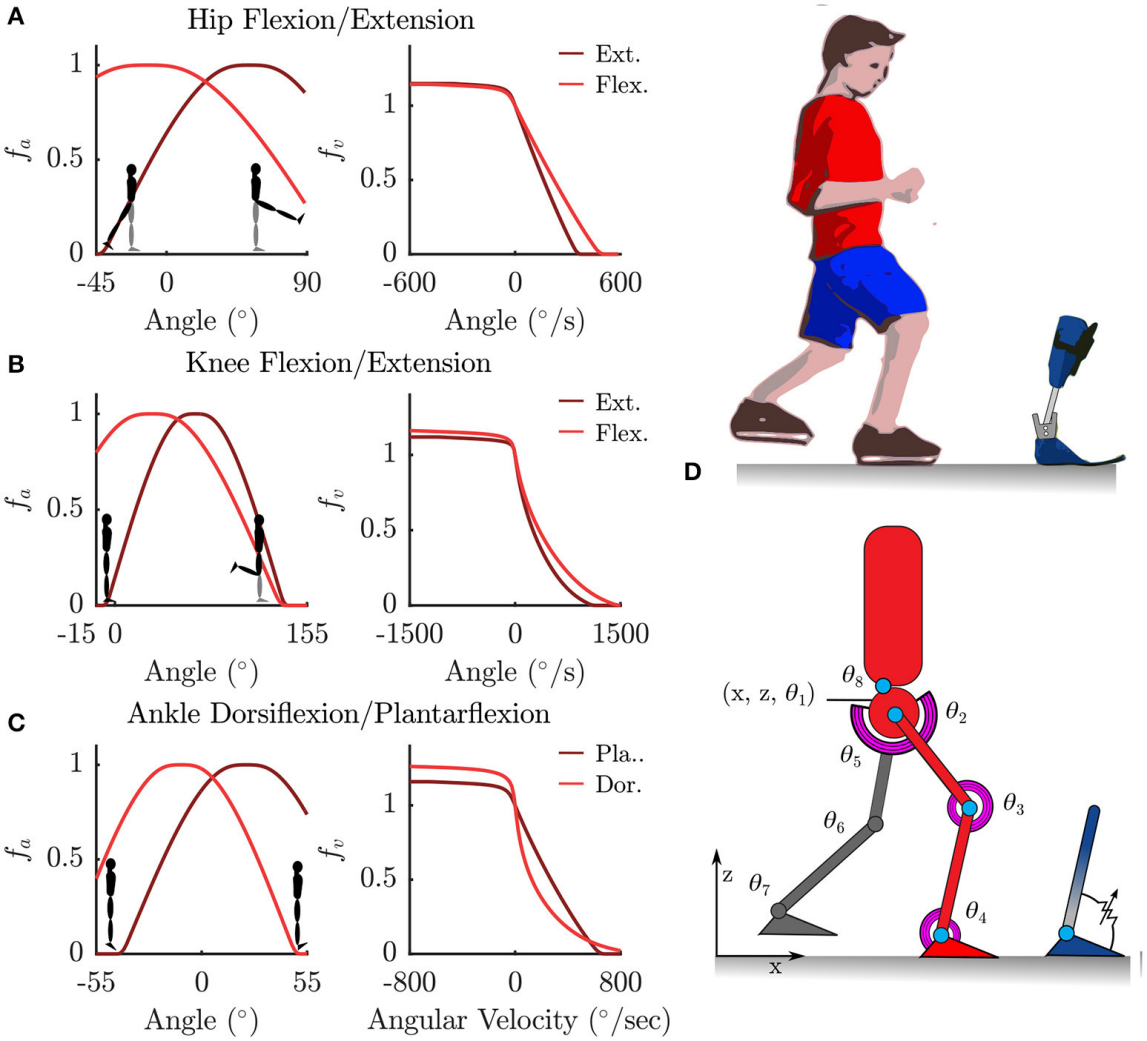
**Torque muscles fitted to the patient were used to actuate a sagittal plane rigid-body model. (A–C)** Show the normalized active-torque-angle curve, **f**^A^(θ), and the torque-angular-velocity curve, **f**^V^(ω), of the torque muscles. **(D)** Illustrates the degrees of freedom of the rigid-body model along with the modeled ankle-foot orthosis as an adjustable-stiffness torsion spring.

We model the human body as an articulated multi-body system with 8 segments, each with one rotational Degree of Freedom (DoF) in the sagittal plane. The pelvis is modeled as a floating base with two additional translational DoFs in the X and Z directions (Figure 1). Segment lengths were approximated from motion capture data, and segment mass and inertia were calculated based on anatomical regression equations for children as per (Jensen, 1986).

#### 2.2.1. Patient-specific muscle torque generator

The rotational DoFs at the hips, knees, ankles and the torso were each actuated by a pair of agonist-antagonist Muscle Torque Generators (MTG), which represent the combined torques being generated by muscle forces in that direction (Figure 1). The active tension developed by a muscle varies non-linearly with the length and contraction velocity of the muscle, while the passive tension varies non-linearly with its length (Zajac, 1988; Millard et al., 2013) (Figure 1). In this study we only model the active components of muscle torque generation. The active torque developed by a MTG varies non-linearly with the angle θ of the muscle and is represented by the normalized *active-torque-angle* curve **f**^A^(θ) which peaks at a torque of τ*Mo* at an angle of θ*o*. During non-isometric contractions the torque developed by the muscle varies non-linearly with the angular velocity ω of the muscle, which is represented by the normalized *torque-angular-velocity* curve ***f***^V^(ω). Muscle torque τ^M^ is computed using these characteristic curves as follows:

(1) τ ​M=τo ​M(af ​A(θ)f ​V(ω))

where *a* is the muscle activation. The active-torque-angle and torque-angular-velocity curves are modeled using C2 continuous Bézier curves (Figure 1) fitted to the experimentally derived torque curves of (Anderson et al., 2007). Anderson et al.'s parameterized curves are not used directly because they are not all C2 continuous, which is required by the OCP solver.

Patient-specific maximum torques in extension for the hip, knee and ankle are estimated under the assumption that during the recorded trials the patient was walking at 90% of his maximum capability (i.e., maximum muscle activations were 0.9). This assumption is motivated by the clinical assessment of this patient's musculature, the pronounced crouch, and slow walking speed observed in the recorded barefoot gait. First, we use inverse dynamics analysis to compute the maximum extension torques generated during the recorded trials. Using *a* = 0.9 and the θ, ω where this maximum occurred, the corresponding maximum muscle torque in extension is found by solving Equation (1) for τ*Mo*. Maximum flexion torques are then computed based on the extension-flexion torque ratios recorded in the study by Anderson et al. (2007). Table 1 lists these values for an age-matched and weight-matched healthy child, as well as for the patient considered in this study. Torso strengths are assumed to be the average of the right and left hip strengths. Note that the MTG models developed here do not take into account the active and passive-dynamic coupling effects of muscles that span multiple joints.

**Table 1:** **Maximum isometric joint torques for an age and weight-matched healthy control and the patient considered in this study**.

	$\tau_{\circ }^{\text{M}}$ **(Nm)**			
	**Healthy**		**Pathological**	
	**Left**	**Right**	**Left**	**Right**
Hip extension	48.82	48.82	30.35	18.64
Hip flexion	34.27	34.27	21.31	13.08
Knee extension	36.08	36.08	24.55	22.40
Knee flexion	19.26	19.26	13.10	11.95
Ankle extension	39.46	39.46	16.84	32.32
Ankle flexion	13.71	13.71	5.85	11.23
Torso extension	48.82		24.49	
Torso flexion	34.27		17.19	

#### 2.2.2. Excitation-activation dynamics

The physiological activation of muscle is an electro-chemical process at the motor unit end plates that converts incoming motor unit action potentials to changes in ion-concentration, and subsequent contraction in muscle fibers. Lumped models provide a simplified representation of this process by relating the overall muscle activation *a*, to the rate of change of activation *ȧ* and neural excitation *e*. Here, we use the formulation by Thelen et al. (2003):

(2) $$\dot{a}=\{\begin{array}{ll} (e-a)\left(\frac{e}{\tau_{\text{A}}}+\frac{1-e}{\tau_{\text{D}}}\right) & \text{if}\;\;e\geq a\\ \frac{e-a}{\tau_{\text{D}}} & \text{otherwise} \end{array}$$

where, τ_A_ = 0.011, τ_D_ = 0.068 denote the activation and deactivation time constants as per (Winters and Stark, 1988).

#### 2.2.3. Parametrized orthosis model

The orthosis worn by the patient consisted of custom-built carbon fiber shank and foot segments joined together by an adjustable-stiffness spring-loaded rotational joint at the ankle. The adjustable stiffness joint was constructed using the Neuroswing Joint (Fior and Gentz, Germany) which needs to be tuned to each patient. The foot segment consist of foot-plate fitted to the patient's foot size and inserted into a standard shoe. The masses of the shank and foot segments are estimated to be 0.34 and 0.69 kg, respectively. Note that the foot segment mass referred to here includes the mass of the shoe. In the following, we refer to the gait with the orthosis+shoe combination as orthosis gait. These masses are added to the shank and foot segments of the patient model for the simulations of orthotic gait.

**Figure 2:**
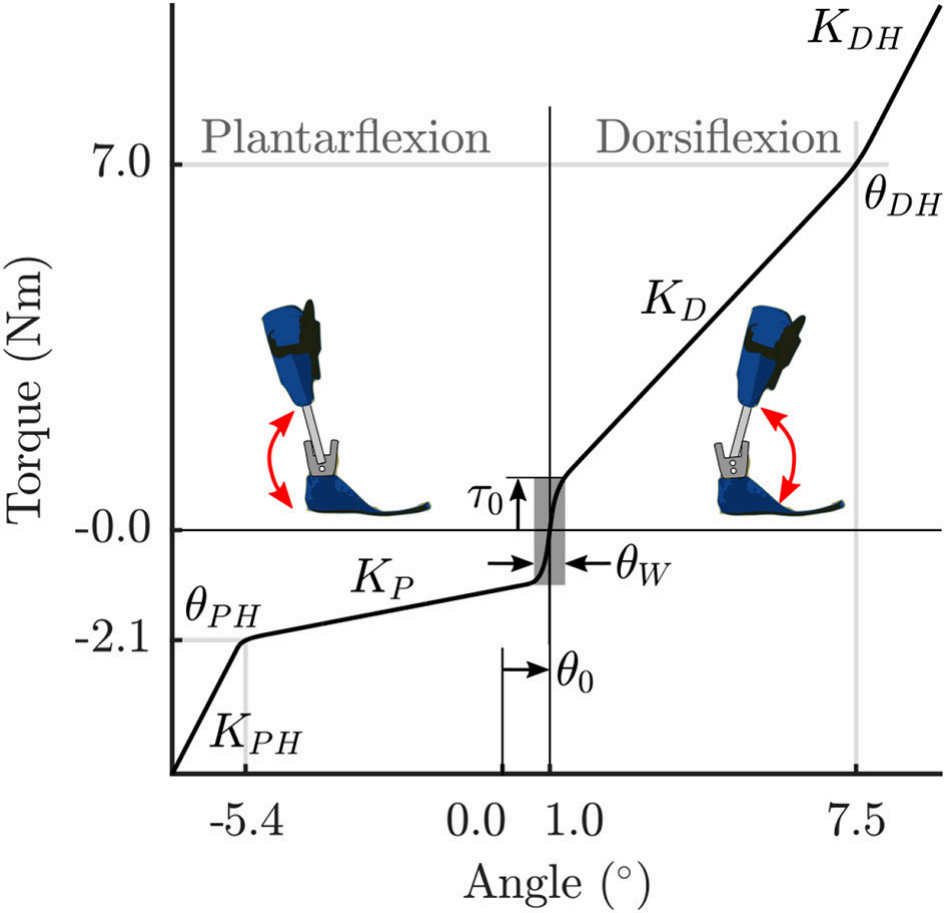
**Parametrized orthosis torque-angle profile: Torques resulting from the stiffness of the orthosis springs and frame are plotted as a function of ankle angle**. The shape of this curve changes as a function of the free parameters *K_D_* and *K_P_*.

The stiffness of the orthosis is modeled as torques generated at the ankle as a function of the ankle angle. The behavior is divided into 5 stages for the extension-flexion range of motion (Figure 2). The parameter θ_0_ defines the offset between the neutral pose of the ankle and the orthosis in a torque-free angular position. In a small angle window θ_*W*_ about this neutral pose, a small pre-load defined by τ_0_ acts on the joint. As the shank rotates with respect to the foot, the torques are produced by the joint springs (spring stiffness *K*_*D*_, and, *K*_*P*_). Upon hitting the adjustable hard stops (θ_*DH*_, and, θ_*PH*_) the shank may rotate further by flexing the carbon fiber material. This relatively stiffer material results in large torques defined by the parameters, *K*_*DH*_ and *K*_*PH*_. The positional parameters listed in Table 2 were measured by the medical professionals during the clinical process. The stiffness of the orthosis-shoe combination is estimated using inverse dynamics analysis of recorded orthosis gait (further details in Section 2.4). While the positional parameters and masses can be measured with a high degree of certainty, the stiffness values of the combined orthosis-shoe unit are tougher to measure and is not part of the clinical routine. In the current approach, we place the initial guess for the stiffness values well below the estimate calculated from the torque-angle characteristics.

**Table 2:** **Orthosis parameters**.

**Parameter**	**Value**
*K*_*DH*_ (Nm/radian)	200
θ_*DH*_ (radian)	−0.12 (L)
	−0.13 (R)
*K*_*D*_^a^ (Nm/radian)	55
θ_0_ (radian)	−0.02
θ_*W*_ (radian)	0.01
τ_0_ (Nm)	±1
*K*_*P*_^a^ (Nm/radian)	5
θ_*PH*_ (radian)	0.09 (L)
	0.1 (R)
*K*_*PH*_ (Nm/radian)	200

a *K_D_ and K_P_ are free parameters of the optimal control problem (OCP) that are to be determined. Values indicated here are the initial guess provided to the OCP. The other parameter values are fixed in the OCP*.

Note that during the clinical fitting/tuning process, the orthotist would adjust the spring stiffness denoted here by the parameters *K*_*D*_, and, *K*_*P*_. While it is possible to adjust the other orthosis characteristics, these springs are the easiest to access and one can quickly test their effects on gait during a fitting procedure. Consequently, we make these two parameters *K*_*D*_, and, *K*_*P*_, free parameters of the OCP that are to be determined. The other parameters in Table 2 are fixed, however, future extensions of this approach could include a more extensive parameter set to be identified. Finally, the overall orthosis torque-angle profile is approximated using C2 continuous Bézier curves that are generated on-the-fly as a function of the changing parameters while running the OCP.

### 2.3. Gait as an optimal control problem

Gait is formulated as a multi-phase OCP, with each phase defined by the attachment and breaking off of sets of contact constraints between the feet and the ground. Due to left-right asymmetry in the patient's gait we model a consecutive left and right stride with *n*_*p*_ = 8 phases as follows (see insets in Figures 4A,B): Right Flat—Left Toe Off, Right Toe On—Right Heel Off, Right Toe On—Left Heel On, Right Toe On—Left Flat, Left Flat—Right Toe Off, Left Toe On—Left Heel Off, Left Toe On—Right Heel On, Left Toe Off—Right Flat. Here, “Flat" indicates that both heel and toe contacts are active. In addition to the position constraints at foot contacts, contact velocities are also constrained to be zero at the start of phases, to ensure continuity in velocities and ground forces. Forces at the load bearing points of the feet are constrained to ensure strictly positive vertical ground reaction forces during the step. Forces in the anterior-posterior direction are constrained to lie within the limiting friction, assuming a coefficient of friction of 0.8 (Chang and Matz, 2001). Forward dynamics computations for the multi-body system subject to the stepping constraint sets are computed using the method described by Kokkevis (2004), implemented in the open-source dynamics library RBDL^1^ by Felis (2017). The OCP then has the general form:

(3) $$\underset{\underline{x}(\cdot ),\underline{u}(\cdot ),\underline{p},\underline{\nu }}{\min}\;\sum_{0}^{n_{p}}\left(\int_{\nu_{j\;-\;1}}^{\nu_{j}}\phi_{j}(\underline{x}(t),\underline{u}(t),\underline{p})dt\right)$$

(4) $$\begin{array}{l} \text{s}.\text{t}.\;\underline{\dot{x}}(t)=f_{j}(t,\underline{x}(t),\underline{u}(t),\underline{p})\text{ for }t\in [\nu_{j\;-\;1},\nu_{j}],\\ \;j=1,...,n_{p},\;\nu_{0}=0,\nu_{n_{p}}=T \end{array}$$

(5) $$0=r_{eq}(\underline{x}(0),..,\underline{x}(T),\underline{p})$$

(6) $$0\leq r_{ineq}(\underline{x}(0),..,\underline{x}(T),\underline{p})$$

(7) $$0\leq g_{j}(t,\underline{x}(t),\underline{u}(t),\underline{p})\text{ for }t\in [\nu_{j\;-\;1},\nu_{j}]$$

where, Equation (3) describes a general objective function to be minimized. Equation (4) is a place-holder that denotes the dynamics of the multi-body system. Note that the actual neuromuscular and multi-body dynamics are described by differential algebraic equations (detailed formulation available in Felis et al., 2015; Felis and Mombaur, 2016; Mombaur, 2016). *x*(*t*) denotes a vector of state variables (generalized coordinates *q*, generalized velocities $\underline{\dot{q}}$, and muscle activations *a*). *u*(*t*) is a vector of control variables (neural excitations *e*). *p* denotes a vector of free model parameters (if any), and, ν is a vector of variable phase switching times with *T* = *t*_*n*_*p*__ = overall time for the motion. Equation (5) denotes coupled and decoupled equality constraints (e.g., switching foot contacts at phase changes), and Equation (6) the inequality constraints (e.g., maintain positive ground reaction force during stepping). Equation (7) denotes all continuous inequality constraints (e.g., bounds of the state variables). Controls *u*(*t*) are subject to constraints formulated in Equation (5) to ensure continuity at phase changes. This is done to ensure 2^*nd*^ order continuity in muscle activations.

To solve the OCP we use a direct multiple-shooting method (Bock and Pitt, 1984) implemented in the software package MUSCOD-II (Leineweber et al., 2003). The direct multiple-shooting approach transforms the infinite dimensional OCP, Equations (4–7), into a finite dimensional non-linear programming problem by first discretizing the continuous controls *u*(*t*) on a grid and then solving the resulting boundary value problem using a multiple-shooting method. Note that with this method the system dynamics are also satisfied between the multiple shooting intervals, leading to physically consistent results throughout the simulated motion. The multi-phase problem described above is discretized into 64 shooting nodes. The controls *u*(*t*) are modeled as piecewise linear functions between discretization points. The works by Felis et al. (2015); Felis and Mombaur (2016) and Mombaur (2016) provide further detail on the constraint formulation, the solution of the multi-body mechanics, and numerical treatment of the OCP. The models and constraints formulation are available as supplementary software code to this article. In our current study we implement four OCPs:

1. LS-Barefoot: Dynamic least-squares fit to recorded barefoot gait
2. MAPD-Barefoot: Minimal activation per distance walked for barefoot gait
3. MAPD-Orthosis: Minimal activation per distance walked for orthosis gait
4. MAPD-WS-Orthosis: Variation of MAPD-Orthosis favoring a higher walking speed

The LS-Barefoot OCP is used to show that our model is capable of tracking the patient's gait in a dynamically consistent manner. Note that we only apply this fitting-type objective function to the recorded barefoot gait, as in a real-world application the gait with orthosis would not be available in advance. The MAPD-Barefoot OCP is used to test how close the chosen cost function can reproduce recorded barefoot gait of the patient. The MAPD-Orthosis OCP is used to predict the patient gait with an orthosis, and simultaneously identify the orthosis spring stiffness parameters. In initial trials we noticed that the predicted walking speed of the MAPD-Orthosis OCP was slower than that of the patient. To further investigate whether our model could be made to walk as fast as the patient, we implemented the OCP MAPD-WS-Orthosis, that contains an additional objective function term favoring a higher walking speed. Note that the OCPs MAPD-Barefoot, MAPD-Orthosis and MAPD-WS-Orthosis are purely synthetic results and no experimental data is used to compute the solutions.

#### 2.3.1. Dynamic least-squares fit to recorded gait

We formulate a fitting-type objective function for the LS-Barefoot OCP that provides a dynamically consistent gait as close as possible to the recorded patient joint kinematics. The objective function is formulated as:

(8) $$\underset{\underline{x}(\cdot ),\underline{u}(\cdot )}{\min}\;\sum_{j=1}^{n_{p}}\left[\begin{array}{l} \sum_{m\;=\;1}^{n_{M,j}}{\left(\underline{q}(t_{jm})-{\underline{q}}^{M}(t_{jm})\right)}^{T}\underline{\underline{W}}(\underline{q}(t_{jm})\\ \;-\;{\underline{q}}^{M}(t_{jm}))+\delta \int_{\nu_{j-1}}^{\nu_{j}}\underline{u}(t)\cdot \underline{u}(t)dt \end{array}\right]$$

Here, the phase times ν are fixed to those obtained from the recorded gait. Note that the generalized coordinates *q*^*M*^ are computed using inverse kinematics at discrete measurement points. $\underline{\underline{W}}$ is a diagonal scaling matrix that may be used to give preference to a closer fit to a subset of the generalized coordinates. Here, we use an identity matrix which provides an overall good fit to all the coordinates. The second term, $\delta \int_{vj-1}^{\text{v}j}\underline{u}\left(t\right)\cdot \underline{u}\left(t\right),$ introduces a small cost that regularizes the control inputs, i.e., it smoothens the control input (neural excitation) and avoids that the solution follows noise in the experimentally recorded data. δ was set to 1*e* − 4 for our computations. For this regularization term all controls are weighted equally relative to each other.

#### 2.3.2. Gait prediction with mapd-type objective functions

We formulate two objective functions for predicting gait: the first minimizes total muscle activations squared per distance walked, and the second contains an additional term that favors a higher walking speed. The first objective function is formulated as:

(9) $$\underset{\underline{x}(\cdot ),\underline{u}(\cdot ),\underline{\nu },\underline{p}}{\min}\;\frac{\sum_{1}^{n_{p}}\int_{\nu_{j-1}}^{\nu_{j}}\underline{a}(t)\cdot \underline{a}(t)dt}{r(T)}$$

Note that dividing by the total distance traveled, *r*(*T*), provides the impetus for moving forward, as without this term the model has no reason to move. Objective functions similar to the one above are commonly used in literature (Thelen et al., 2003; Damsgaard et al., 2006; Ackermann and van den Bogert, 2010) and are associated with the minimization of muscle effort (Ackermann and van den Bogert, 2010). We introduce additional periodicity constraints on all the state variables and the controls, such that the initial states at the start of the first phase matched the final states at the end of the last phase.

The second objective function includes a term favoring a higher walking speed and is formulated as:

(10) $$\underset{\underline{x}(\cdot ),\underline{u}(\cdot ),\underline{\nu },\underline{p}}{\min}\;\frac{\sum_{1}^{n_{p}}\int_{\nu_{j-1}}^{\nu_{j}}\underline{a}(t)\cdot \underline{a}(t)dt}{r(T)}-\lambda \frac{r(T)}{T}$$

where, λ is a scaling term. The objective function (Equation 9) is used in the OCPs MAPD-Barefoot and MAPD-Orthosis. The objective function (Equation 10) is used in the OCP MAPD-WS-Orthosis. For the OCPs MAPD-Orthosis and MAPD-WS-Orthosis, there are 4 free parameters to be determined during the optimization. These corresponded to the left and right pairs of orthosis spring stiffness parameters (*K*_*D*_,*K*_*P*_). The orthosis dynamics in these OCPs are simulated using the values listed in Table 2.

### 2.4. Evaluation procedure

We evaluate the model and the predicted results in the following ways:

1. We report the residuals from inverse dynamics analysis of the recorded data. Inverse dynamics analysis computes generalized forces that are consistent with the kinematics of the patient and the measured ground forces. Since our kinematic model has a floating pelvis frame the inverse dynamics results will include residual forces: the generalized forces between the ground frame and the pelvis frame. If these residual forces are small in magnitude then we can conclude that the geometry and mass distribution of the model fits the subject well.
2. We use the LS-Barefoot formulation to assess the quality of the foot-ground contact model. This is because, although the objective function is trying to drive the model to walk with the same kinematics as were used in the inverse dynamics analysis, the foot-ground constraints must be satisfied. Any differences that show up between the LS-Barefoot results and the recorded gait can be ascribed to how well the model of foot-ground contact fits the patient.
3. We compare the solution of MAPD-Barefoot to the kinematics and kinetics of patient to assess how well our chosen cost function fits the movement of subject.
4. We evaluate the predicted orthosis parameters and subject gait by comparing the solution of MAPD-Orthosis to the corresponding experimental data. Any new differences that appear between the OCP results and the experimental data are either due to differences between our orthosis model and the real orthosis, or because the patient no longer walks in a manner that is consistent with our chosen cost function.
  To separate these differences, we compare the net torque-angle profile of the MAPD-Orthosis results to the corresponding experimental data. If the net ankle torque-angle profiles are similar it is likely that the remaining differences we observe are happening because the patient is no longer walking in a manner that is consistent with our chosen cost function. It is necessary to use the net ankle torque (the sum of the torque contribution of the ankle MTGs and the orthosis) in this comparison because the kinematics and kinetics of the patient's ankle were not recorded separately from the orthosis.
5. We perturb the free orthosis parameters by −5% in the vicinity of the identified optimal values to compute how the cost function value, knee flexion angle (and thus severity of crouch), step lengths and walking speed vary with the stiffness of the orthosis.

## 3. Results

**Figure 3:**
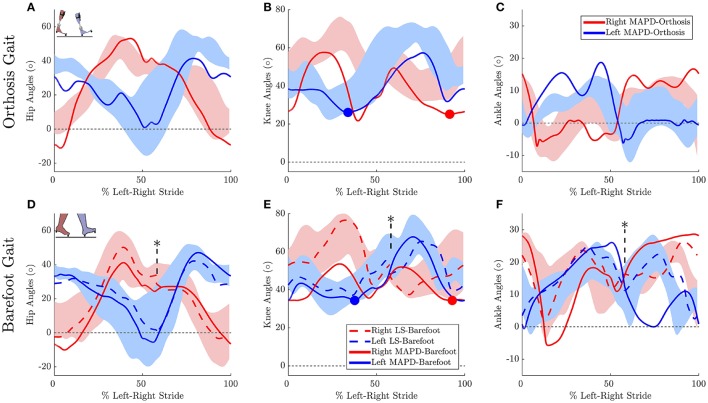
**Gait kinematics: Top panels plots the joint angles for orthosis gait for the (A)** hip, **(B)** knee, and **(C)** ankle joints. Solid lines plot the solution of the MAPD-Orthosis. Shaded areas indicate the range of the recorded patient joint angles. **(D–F)** plot the corresponding results for MAPD-Barefoot (solid lines), and the results from the LS-Barefoot dynamic fit (dashed lines). Note that the LS-Barefoot results have a discontinuity as indicated by asterisks on **(D–F)**. This is due to the difference between the setup of the optimal control problem (starting at left toe off), and the plots (starting at left heel strike), as well as an asymmetry in the patient's gait over one complete left-right stride. We denote 100% along x-axis as the full left and right stride. Insets in panels **(A,D)** indicate the starting pose of the right and left foot. Filled circles in panels **(B,E)** indicate the minimum knee angle during stance.

The residual forces from the inverse dynamics analysis for barefoot and orthosis gait are under 3.3 N in the anterior-posterior and vertical directions while the sagittal plane moments are under 0.12 Nm (Table 3). The kinematics of the LS-Barefoot solution closely matches the patient's barefoot gait kinematics (dashed lines in Figures 3D–F), with RMS differences of 0.83° at the pelvis, 1.52° at the hips, 2.43° at the knees, and 2.64° at the ankles. The ground reaction forces of the LS-Barefoot solution deviate from the patient's recorded ground reaction forces with RMS differences of 68.24 N in the vertical direction. Note that all RMS differences are computed with respect to the average corresponding recorded gait kinematics and ground reaction forces.

**Table 3:** **Residuals from inverse dynamics analysis**.

		**Mean**	**Min**	**Max**
Barefoot	A-P (N)	−0.02	−0.27	0.16
	Vert. (N)	1.36	−0.04	2.8
	Mom. (Nm)	−0.02	−0.11	0.09
Orthosis	A-P (N)	−0.05	−0.16	0.01
	Vert. (N)	1.6	0.0	3.28
	Mom. (Nm)	−0.05	−0.15	0.11

*A-P denotes the forces in the anterior-posterior direction, Vert. denotes the forces in the vertical direction, Mom. denotes the moments about the free flier joint*.

**Figure 4:**
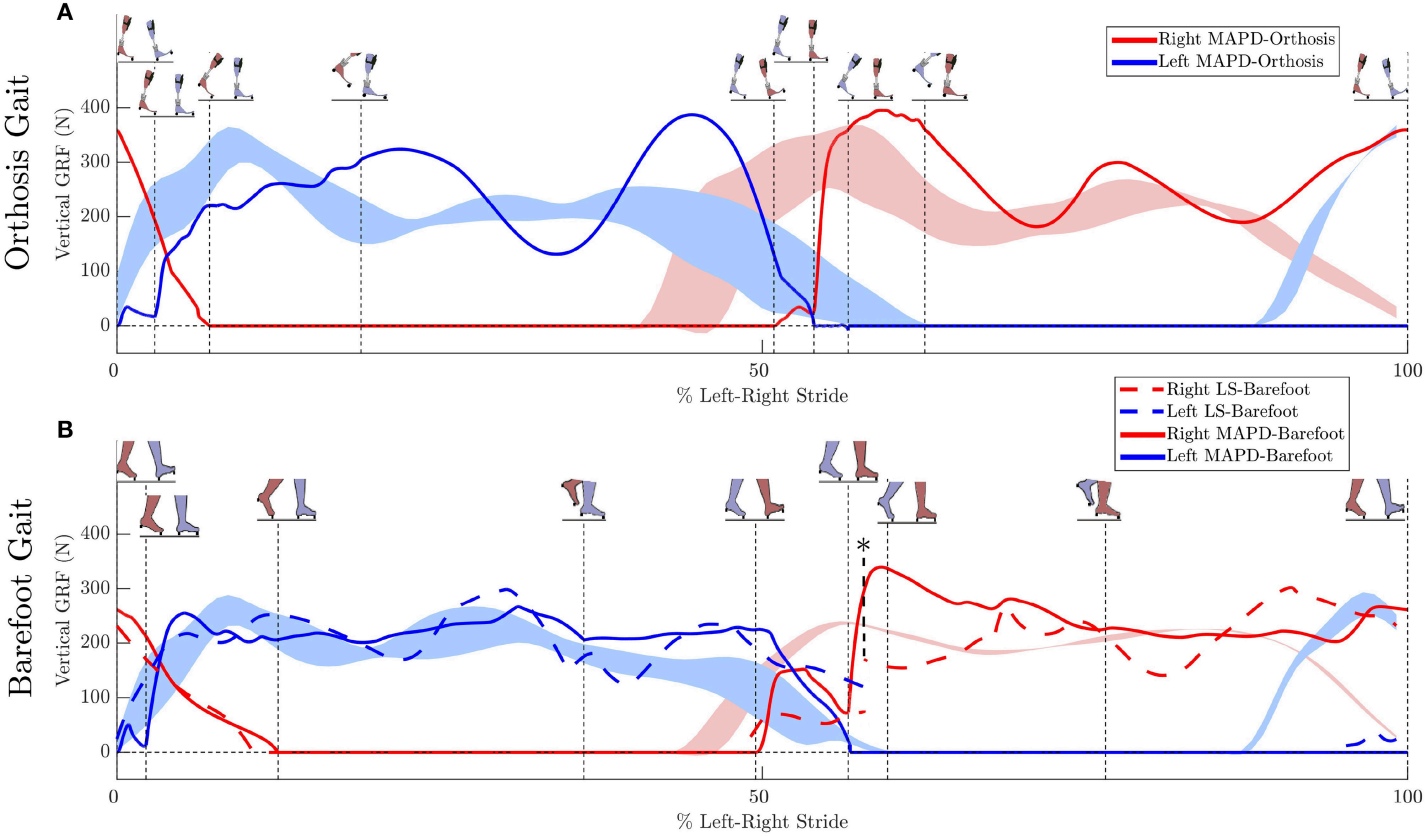
**Ground reaction forces (GRF): (A)** Solid lines plot the simulated GRFs for MAPD-Orthosis gait. **(B)** GRFs for MAPD-Barefoot gait. Dashed lines indicate results for the LS-Barefoot gait. Shaded areas indicate the range recorded. Vertical dashed lines indicate the phase changes (foot contact events as shown in the figure insets). We denote 100% along x-axis as the full left and right stride. Note that the LS-Barefoot results have a discontinuity as indicated by the asterisk. This is due to the difference between the setup of the optimal control problem (starting at left toe off), and the plots (starting at left heel strike), as well as an asymmetry in the patient's gait over one complete left-right stride.

The MAPD-Barefoot gait step lengths and walking speed were within the range recorded on the patient (Table 4). The kinematic differences were larger when compared to LS-Barefoot, with RMS differences of 7.57°, 12.95°, 13.67°, 12.22°, for the pelvis, hip, knee and ankle angles respectively. To put these kinematic differences in perspective, note that the patient walks with a high degree of variability, exhibiting maximum variances in the barefoot trials of between 5.5° and 13.34°. The RMS values of the MAPD-Barefoot ground forces are 81.1 N. The MAPD-Barefoot problem took 4 h to solve as a single-thread execution on a 3.6 GHz processor.

**Table 4:** **Comparison of recorded gait characteristics and results from the corresponding optimal control problems**.

	**Step length (m)**		**Walking speed (m/s)**
	**Left**	**Right**	
Recorded range barefoot	0.30– 0.43	0.22–0.37	0.51–0.73
MAPD-Barefoot	0.37	0.29	0.60
Recorded Range	0.40–0.47	0.34–0.46	0.70–0.98
MAPD-orthosis	0.32	0.41	0.62

The MAPD-Orthosis model walked with a lower cost, less of a crouch, a longer right step, and a higher walking speed than the MAPD-Barefoot trial (Table 4). Compared to the MAPD-Barefoot gait the MAPD-orthosis gait extends its right and left knees 9.26° and 8.3° more during stance, respectively (indicated as filled circles in Figures 3B,E). This improvement in knee extension angles matched the trend seen in the patient recordings. The RMS differences for MAPD-Orthosis gait are 8.48° at the pelvis, 14.48° at the hips, 16.11° at the knees, and 8.28° at the ankles (Figures 3A–C). The RMS differences for ground-reaction forces are 128.04 N for MAPD-Orthosis, which is higher than that for MAPD-Barefoot gait. Even though the orthosis pushed the model to walk faster, the left step length and the walking speed are below the corresponding recorded ranges (Table 4). The OCP MAPD-WS-Orthosis with the modified objective function, Equation (10) and a λ = 2, results in a walking speed of 0.77 m/s which is within the recorded range. The MAPD-Orthosis problem took 7 h to solve as a single-thread execution on a 3.6 GHz processor.

**Figure 5:**
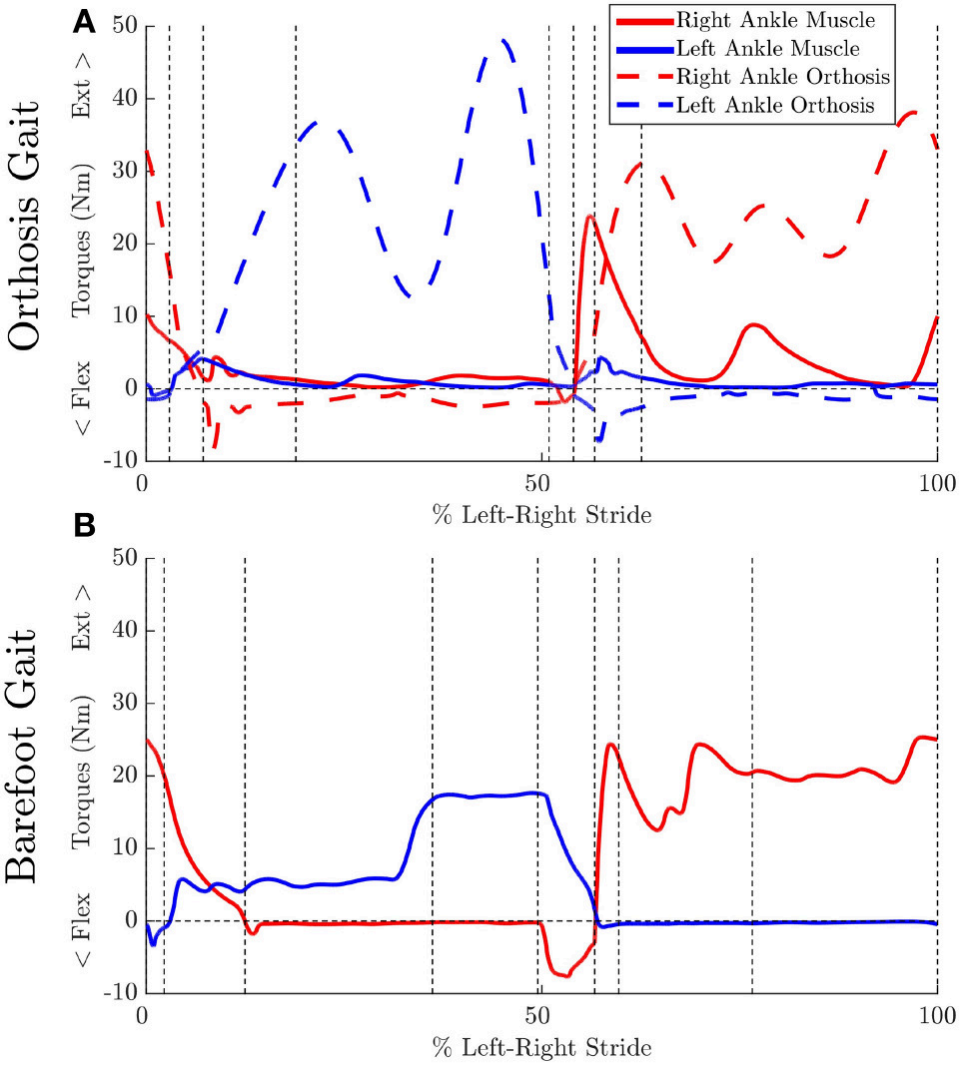
**Effect of orthosis on MTG torques (extension + flexion) at the ankle: Vertical dashed lines indicate the phase changes (A)** MAPD-Orthosis gait. Dashed lines indicate torques generated by the orthosis. **(B)** MAPD-Barefoot gait.

**Figure 6:**
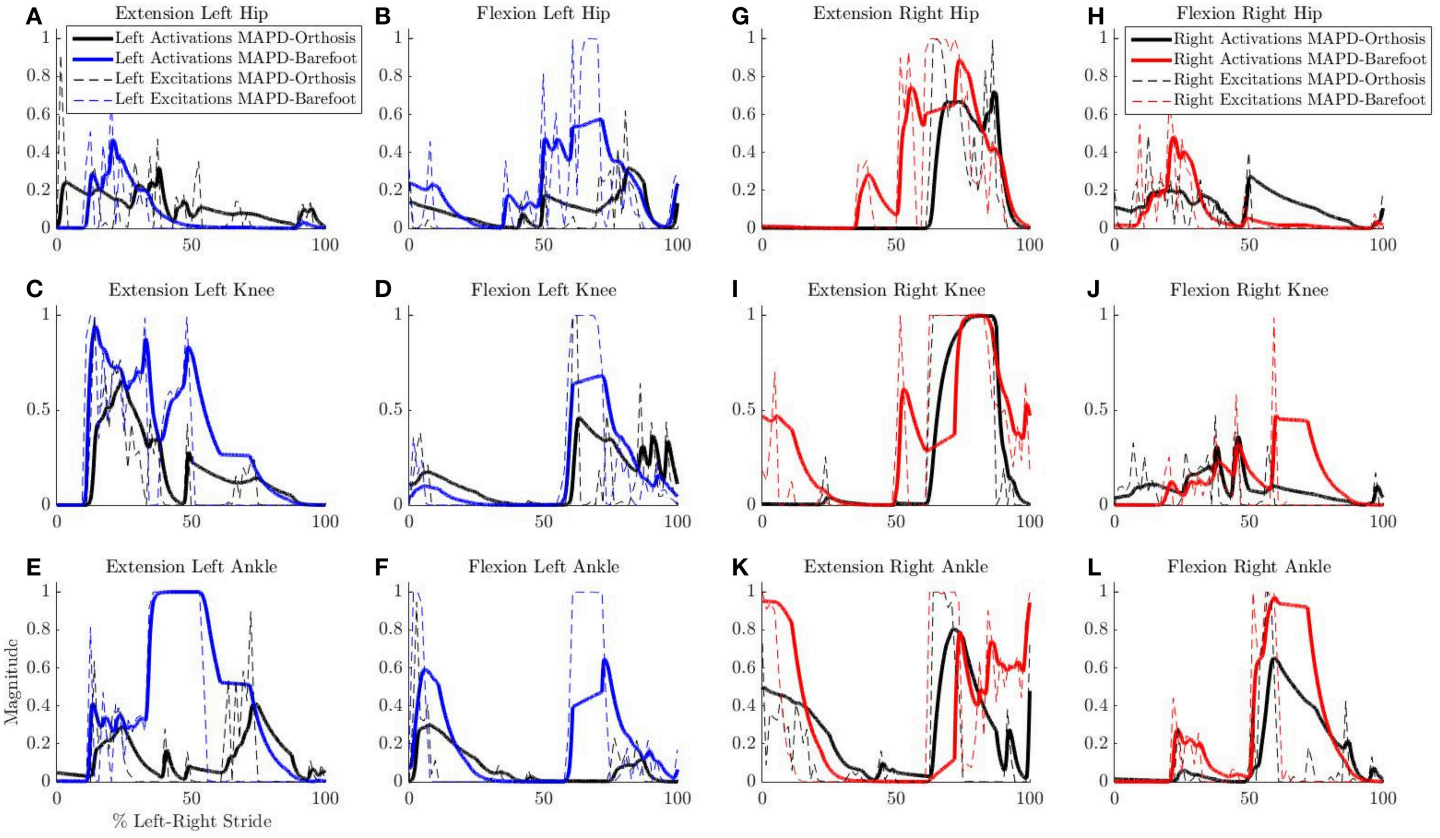
**Comparison of neural excitations ***e*** and muscle activations ***a*** for MAPD-Orthosis and MAPD-Barefoot gait**. Note that the two simulations resulted in different overall durations and are presented here with respect to % left-right stride. **(A–F)** Plot the results for the muscles of the left lower limbs, and **(G–L)** those for the right lower limbs.

Ankle muscle extension torques are substantially reduced for the MAPD-Orthosis gait compared to those for MAPD-Barefoot (Figures 5A,B). Despite the faster walking speed for orthosis gait, the corresponding activations and excitations are generally smaller or equivalent to those for MAPD-Barefoot (Figure 6). Overall the objective function cost for the MAPD-Orthosis is smaller than that for MAPD-Barefoot (0.61 and 2.2, respectively). The computed optimal orthosis spring stiffness are *K*_*D*_ = 45.9 Nm/rad and *K*_*P*_ = 13.2 Nm/rad for the right ankle orthosis, and *K*_*D*_ = 62.8 Nm/rad and *K*_*P*_ = 19.7 Nm/rad for the left ankle orthosis.

**Figure 7:**
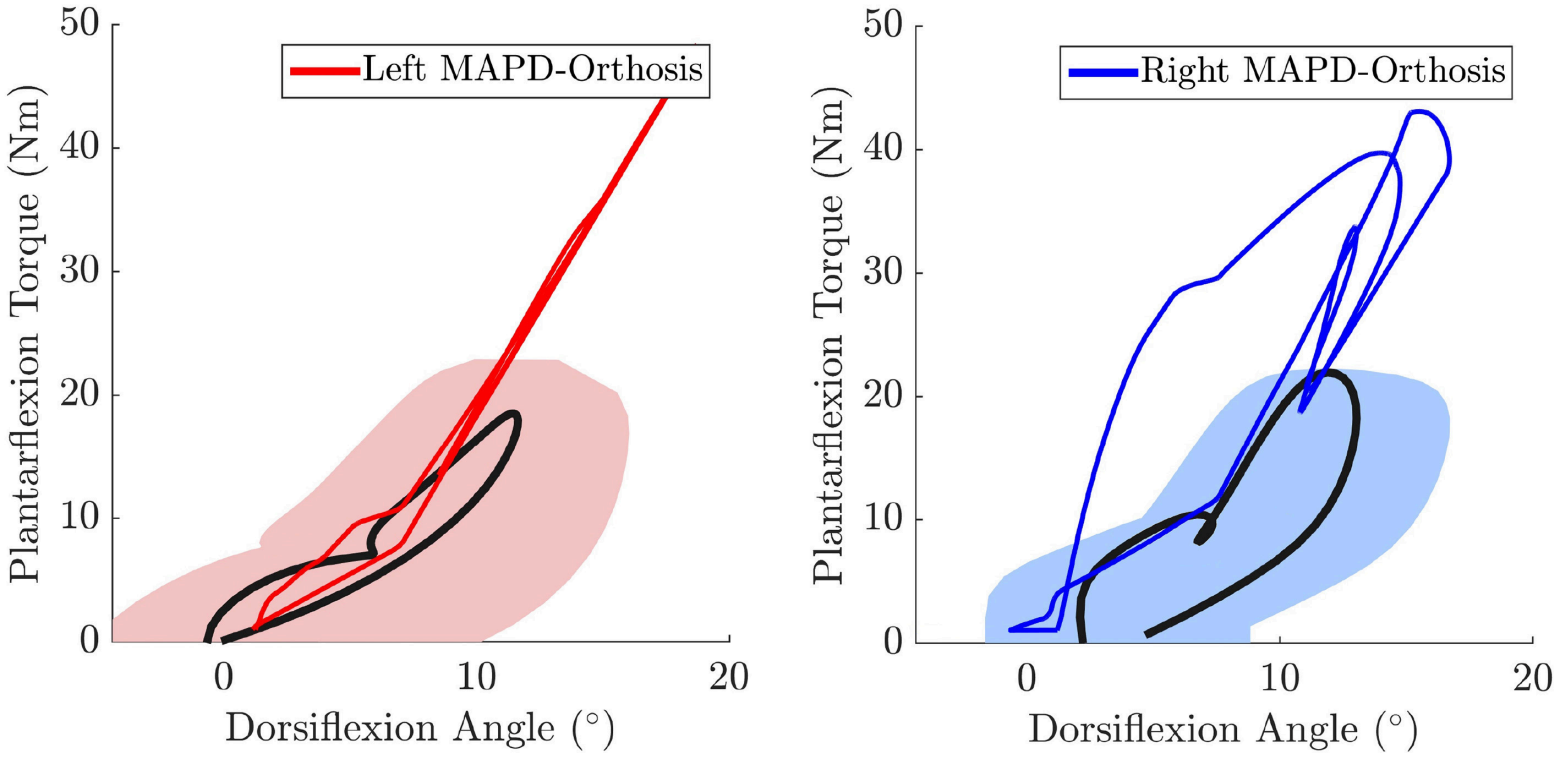
**The MAPD-Orthosis net torque-angle profiles (solid red and blue lines) are plotted against the mean torque-angle profile of the patient (solid black line) and the area that encompass ± 1 standard deviation (shaded regions)**. Note that the torque that is plotted is the sum of the net MTG torques (extension + flexion) and the orthosis torque. This torque is equivalent to the torque computed by the inverse dynamics analysis of the patient when he is wearing the orthosis.

The net torque-angle profiles of the MAPD-Orthosis gait have a similar angular offset and slope to the mean torque-angle profiles of the patient (Figure 7). Though the peak torques of the MAPD-Orthosis gait are larger than those of the patient, the profiles overlap with the ± 1 standard deviation regions of the patient data (shaded regions). The average slope of the torque-angle profiles from the patient data range from 95.7 to 135.5 Nm/rad, while the slope of the MAPD-Orthosis torque-angle profiles range from 122.3 to 150.1 Nm/rad.

The perturbation analysis reveals a maximum difference of 1.75% in cost function value, min. knee angles, step lengths and walking speeds for −5% changes in the orthosis stiffness parameters (Table 5).

**Table 5:** **Results from the parameter perturbation analysis**.

**Perturbed parameter**	**Perturbation size**	**Φ**	**Min. knee angle**		**Step length**		**Walking speed**
			**Left**	**Right**	**Left**	**Right**	
Left *K*_*D*_	−5%	1.75	−0.32	−1.14	0.30	0.35	−0.002
Left *K*_*P*_	−5%	1.10	−0.65	−0.11	−0.26	0.68	0.39
Right *K*_*D*_	−5%	0.11	0.08	−1.39	0.03	0.27	−0.06
Right *K*_*P*_	−5%	0.67	−1.02	0.87	0.09	0.89	0.56

*A −5% perturbation was applied to each of the optimal identified spring stiffness values. Rows indicate the % change in MAPD-Orthosis results for a change in the corresponding orthosis spring parameter. Φ Denotes the cost function value (Equation 9). A negative % change in the min. knee angle indicates a straightening of the knee during stance*.

## 4. Discussion

We have presented an optimal control approach to generate novel movements with physically consistent dynamics and applied it to the simulation of patient gait. Our simulations result in smooth ground reaction forces (Figure 4) as well as muscle torques (Figure 5), and can be computed with modest computational resources. The ground reaction forces are continuous and have similar shape and magnitude as the patient observations. This is an improvement from published literature in this field, where large transients as well as deviations upto 150 to 200% of body weight have been reported (Ackermann and van den Bogert, 2010; Dorn et al., 2015). Physiologically realistic ground reaction forces are important, because a discrepancy here propagates through the model resulting in unrealistic joint torques and muscle forces. These characteristics, along with the possibility of tuning the model parameters to reflect weakened muscles, are important first steps toward applying such methods in clinical settings.

The low residual forces from the inverse dynamics analysis (Table 3) indicates that the geometry and mass distribution of the model fit the patient well^2^. For comparison these residual values are 1.0% of the peak ground reaction forces during walking. The LS-Barefoot results reveal that the model is able to follow the recorded patient kinematics with the RMS differences smaller than the stride-to-stride variation in the patient. However, the ground reaction forces are markedly less smooth (dashed lines in Figure 4B) when compared to those recorded from the patient. In contrast the model kinematics for MAPD-Barefoot show larger RMS errors than LS-Barefoot, however, the ground reaction forces are smoother. We also note that the duty factor (ratio stance vs. swing time) in our simulated gait is different from that recorded, with shorter double stance durations for MAPD-Orthosis (Figure 4A).

Taking these results together, we conclude that the most likely reasons for these differences are the shape of the foot and the enforced sequential nature of the contact phases. Modeling the foot as a flat surface simplifies the resolution of the contact dynamics, however, it overlooks the natural curvature of the foot and the associated influence this can have on the behavior of the rest of the body (Dorn et al., 2012). Foot contact dynamics has been recognized as an issue of significant importance in model based estimation and prediction of gait as the foot forces affect those at the hip, knee and ankle (Dorn et al., 2012; Millard and Kecskeméthy, 2015). The use of a suitable curved foot model would therefore help improve the contact dynamics as well as avoid the strict phases that we have imposed in our current formulation. We expect that a curved foot model would also improve the simulated kinematics of the knee and ankle, which currently show large deviations from recorded behavior.

The orthosis provides additional ankle torque especially during push-off, and the resulting orthosis-equipped model could walk faster, with more extended knees than the barefoot model. The slope of the torque-angle profile of the MAPD-Orthosis is close to that of the patient (Figure 7). This indicates that the identified orthosis stiffness values are likely close to those of the patient's orthosis. We recall that the patient's orthosis was manually tuned by the orthopedic professional during the clinical procedure. We remark that while the slopes and angular offsets of the orthosis-equipped model lie within the experimentally recorded variation, the magnitude of the torques were higher in the model. This indicates that either the foot-shape (lever arm during toe-off) or the cost-function need to be updated to better match the patient. Our perturbation analysis reveals a systematic increase in the cost function value (which is consistent as the perturbation was applied about the optimal solution) and relatively small influence of parameter changes on the gait characteristics (Table 5).

Despite the higher walking speed of the orthosis gait, the overall distance-normalized muscle activations based cost is smaller than that for barefoot gait. We observe a strong reduction in the muscle activations for orthosis walking (Figure 6). Although this is a desirable effect as it points toward a less fatiguing gait, it is presently unclear whether these changes actually occurred in the patient's real muscular efforts. As we are missing the experimental EMG recordings for this gait, our simulated reduction in muscle activations must be viewed as plausible but unverified. As noted in our Introduction, this is a general open problem with neuromuscular models, which require further experimental efforts as well as technological advances in EMG technology.

### 4.1. Choosing an optimality criterion for gait

Our simulations are driven by an optimization criteria that minimizes the square of muscle activations per distance walked. Higher powers of activations have been suggested to be associated with muscle effort (Ackermann and van den Bogert, 2010), and our results from MAPD-Barefoot show that this formulation provides a reasonable match to the recorded gait characteristics (Table 4). For orthosis-equipped gait, we observe that the same formulation (MAPD-Orthosis), resulted in gait that is slower and has smaller steps. With an additional term in MAPD-WS-Orthosis we could drive the simulation toward more desirable characteristics, in this case faster walking. We speculate that there are subtle differences in the patient's walking behavior with orthosis, that are not entirely covered by the MAPD-only formulation.

Note that an alternative explanation for the slow walking speed in MAPD-Orthosis could lie in an underestimation of the maximum isometric torques of the patient's muscles, as well as the missing torques provided by the passive musculotendon components. We explored this avenue by simulating gait of a healthy age-matched, weight-matched child (torque values listed in Table 4). The detailed plots are provided in the supplementary section to this article. With healthy muscle strengths, we observed that the model was capable of longer steps and faster walking speed, matching the recorded gait of typically developing children (Schwartz et al., 2008). This leads us to believe that the major reason for the slower gait in MAPD-Orthosis lies in the cost function formulation, and that this deserves further investigation. For example with the use of inverse optimal control methods to identify the particular cost function that best describes experimentally recorded behavior (Mombaur, 2016), and especially the specification of cost functions that are better suited for pathological gait.

From our current work, we show that the specification of muscle strength in our models and the MAPD-type objective function is capable of reproducing, at least in our case study, a range of walking behaviors from healthy to pathological. Overall, it is foreseeable that a generic class of such objective function terms may be made available to the medical specialist, that would correspond to the clinical goals for the patient (e.g., faster walking, less crouch, reduced movement of the center of pressure etc.). The ultimate decision on which of these characteristics are suitable for the patient, would be the responsibility of the orthotist and other medical professionals. Our methods could provide a virtual window into the expected behavior under these conditions without inconveniencing the patient.

### 4.2. Limitations and perspectives

In addition to the shape of the foot, we believe that another improvement to the model would be to decouple the orthosis and body models. This would allow for a more realistic simulation of the body-orthosis interaction as well take into account the inertial effects of the orthosis independently from the body. Specifically, this decoupled formulation would enable us to calculate a comfort-like cost function term based on the contact forces being generated, and as well simulate the effects of non-aligned rotations between the foot and the orthosis. Together, we believe that these changes will contribute toward more natural looking behaviors in our synthesized gait.

The simulated activations and active muscle forces of our model may be further improved. We estimated the patient muscle strengths based on inverse dynamics analysis and a qualitative clinical assessement of how close the patient was to his maximum strength during the recorded gait. The muscle curves used in our MTG model come from (Anderson et al., 2007), that are based on adult subjects. These curves may look different from children, especially for those with a pathology that affects the muscle and overall strength. To the best of our knowledge, no such quantative muscle studies exist for children, and it would be of interest to bridge this gap in experimental data in the future. In a general context, the accurate specification of the model to a person is still an open problem. There may be various approaches to solve this, for example by using direct dynamometry information when available, and/or by making the maximum isometric torque as parameters of an OCP. Future iterations of this approach would include passive musculotendon forces in the simulations. To this end we are evaluating methods to estimate passive forces and muscle model coefficients from experimental data. Additionally, modeling the effects of muscles that span multiple joints is an important next step. This may be implemented as a combination of the MTGs used in this work, and some of the major anatomical muscles as line-type models. For the study of pathological gait this may be especially important, as it would then allow the freedom to include the more complicated line-type muscle models based on the specific question/pathology at hand. In this initial work we do not model the feedback dynamics of muscle reflexes like for example those in the work by Geyer and Herr (2010). Including these closed-loop dynamics makes the OCP harder to solve, and we are currently exploring formulations that work well with our framework. While reflexes are typically subdued during normal locomotion (Brooke et al., 1991), they play an important role in making gait robust against perturbation rejection. In addition, neuromuscular pathology can adversely affect the ability to modulate reflexes (Hodapp et al., 2007; Pearson and Gordon, 2013), and any implementation of reflex feedback for pathological gait would necessarily require more detail and study than currently available in the state of the art.

Finally, we have focused so far on movements in the sagittal plane and used a case study to provide an important proof-of-concept of our methods. Our comparison to experimental data provides a first evaluation of our model and technical platform, that needs to be further validated with a prospective clinical trial and extended to include movements in the transverse plane. We acknowledge that for application in a clinical setting our methods would also need to allow an easy setup and tuning to individual patients. Note that although the setup of the OCPs in this work took a significant amount of time, these efforts do not need to be replicated for each patient. In a future clinical application, we envision that a standardized gait simulation may be solved within a few hours with an individualized patient model (which requires relatively little time). This scenario provides a realistic means to apply our methods in a true clinical setting, and would be the ultimate goal of our future efforts related to this work.

## Author contributions

MS, MM, and SW designed the study. MS, MM, MF, and KM performed the computational work related to this study. All authors contributed to the interpretation of the data and in preparing the manuscript.

## Funding

This study was part of the Frontier-Orthosis project supported by the German Excellence Initiative within the third pillar funding of Ruprecht-Karls-Universität Heidelberg. We acknowledge financial support by Deutsche Forschungsgemeinschaft and Ruprecht-Karls-Universität Heidelberg within the funding programme Open Access Publishing.

### Conflict of interest statement

The authors declare that the research was conducted in the absence of any commercial or financial relationships that could be construed as a potential conflict of interest.

## Footnotes

^1^https://rbdl.bitbucket.io

^2^http://simtk-confluence.stanford.edu:8080/display/OpenSim/Simulation+with+OpenSim+-+Best+Practices

## Supplementary material

Additional results figures are available as Supplementary Material to this article. The patient data, models, and OCP formulations are available as Supplementary Data. Further developments of this work will be maintained as an open-source public repository.^3^
