# Supplementary material for: Optimal Control Based Stiffness Identification of an Ankle-Foot Orthosis Using a Predictive Walking Model
Source: Front Comput Neurosci. 2017 Apr 13;11:23. doi: 10.3389/fncom.2017.00023 (PMC5390028; doi:10.3389/fncom.2017.00023)
Supplement: Supplementary file 2 [file Image1.pdf]

Supplementary Material: Optimal control based stiffness identification of an ankle-foot orthosis using a predictive walking model

Manish Sreenivasa*, Matthew Millard, Martin Felis, Katja Mombaur, and Sebastian I. Wolf

*Correspondence:
Author Name: Manish Sreenivasa
manish.sreenivasa@iwr.uni-heidelberg.de

1 SIMULATION OF HEALTHY GAIT

We simulated walking, MAPD-Healthy, by building a model with the muscle parameters of a typically developing control subject. Joint lengths and segment mass/inertia were matched to the recordings from a 7-year old typically developing child (height 1.26 m, weight 25.9 kg). The model's left-right limbs were symmetric and we therefore only computed the left stride in the optimal control problem (OCP) (the results below show the symmetric solution mirrored for the right side limbs). The OCP objective function minimized muscle activations per distance walked (MAPD) (see main paper for details). We compare the MAPD-Healthy results to the typical gait characteristics of a 10-year old as reported by Schwartz et al. (2008).

Results:

The model walked with a step length of 0.44 m and walking speed of 0.93 m/s which are close to the ranges reported in Schwartz et al. (2008) of 0.44-0.50 m and 0.99-1.12 m/s, respectively. The model also produced similar kinematics and ground reaction forces as those reported in typically developing children (Fig. S1).

REFERENCES

Schwartz, M. H., Rozumalski, A., and Trost, J. P. (2008). The effect of walking speed on the gait of typically developing children. *Journal of Biomechanics* 41, 1639–1650

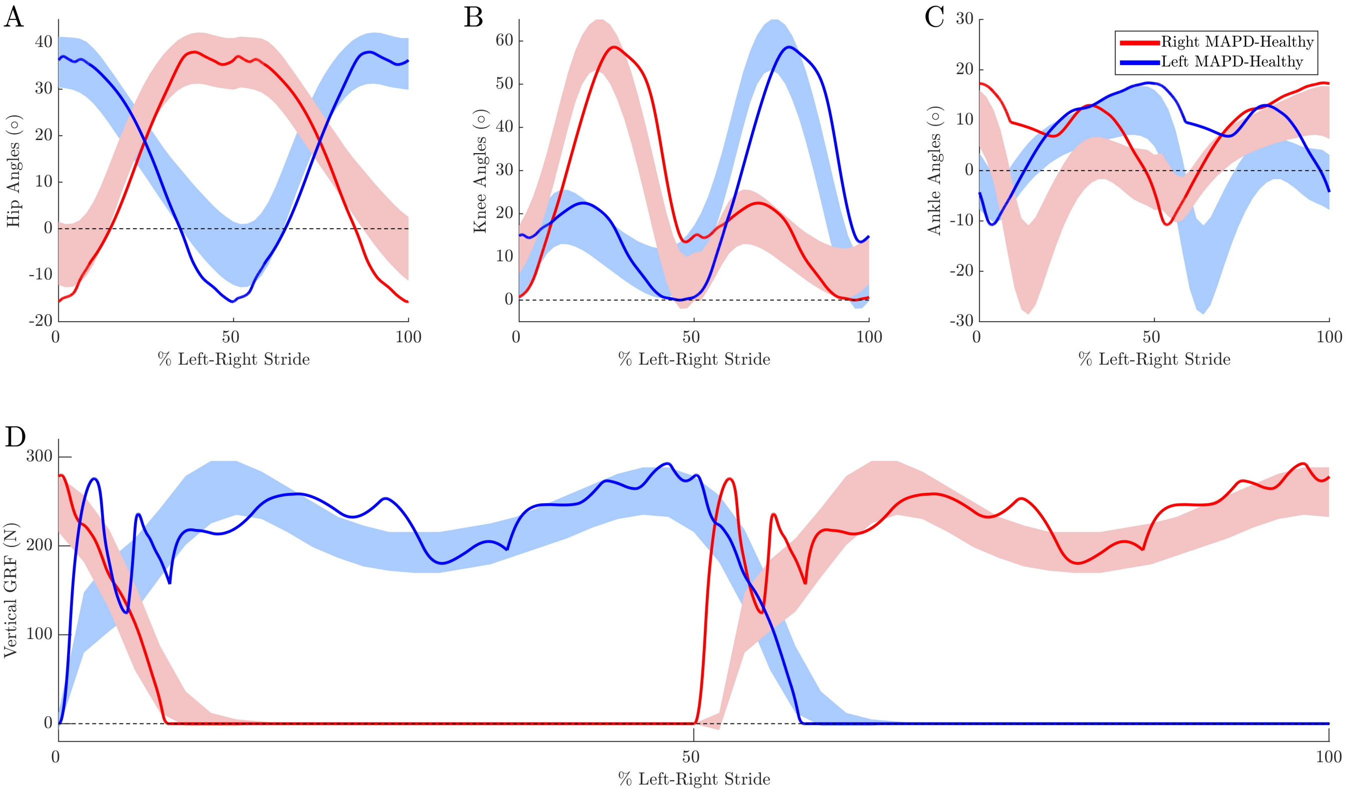

Figure S1. Results from MAPD-Healthy: Top panels plots the joint angles for the (A) hip, (B) knee, and (C) ankle joints. Panel (D) plots the ground reaction forces. Shaded regions indicate the variance reported by (Schwartz et al., 2008) for a 10-year old typically developing child.
