# Supplementary material for: Optimal Control Based Stiffness Identification of an Ankle-Foot Orthosis Using a Predictive Walking Model
Source: Front Comput Neurosci. 2017 Apr 13;11:23. doi: 10.3389/fncom.2017.00023 (PMC5390028; doi:10.3389/fncom.2017.00023)
Supplement: Supplementary file 1 [file DataSheet1.ZIP › PathWalker/PathWalker-CodeDescription.pdf]

PathWalker Distribution
Description of OCP software code

Supplementary material for the study "Optimal control based stiffness identification of an ankle-foot orthosis using a predictive walking model", by M. Sreenivasa, M. Millard, M. Felis, K. Mombaur & S.I. Wolf
Contact: M. Sreenivasa <manish.sreenivasa@iwr.uni-heidelberg.de>,
Heidelberg University, Germany

This document briefly describes the software code that setups the OCPs. Please note that the c++ code is not meant to be compiled as the dependency Muscod is not available as open-source code.

The following OCPs are described

- LS-Barefoot
- MAPD-Barefoot
- MAPD-Orthosis
- MAPD-WS-Orthosis

In each of the corresponding “code” folders the following files are provided

- pathWalker2d.cc
  Setup of the constraints and optimal control problem
- pathWalker2d_Model.cc & pathWalker2d_Model.h
  Setup of the model dynamics
- pathWalker2d_Enums.h
  Lists the states, controls and parameters for the OCP

Code description LS-Barefoot OCP

In LS-Barefoot/code/pathWalker2d.cc: Objective functions lsqfcn_trackMotion and lfcn_reg are the tracking and regularization functions respectively:

```
// Objective function (Lagrangian type)
void lfcn_reg (double *t, double *xd, double *xa, double *u,
                    double *p, double *lval, double *rwh, long *iwh, InfoPtr *info) {
  unsigned int i;
  *lval = 0.;
  double regFactor = 1e-3;
  for (i = 0; i < NoOfControls; i++) {
    *lval = u[i]*u[i];
  }
  *lval*=regFactor;
}
// Objective function (Least-Squares)
void lsqfcn_trackMotion (double *t, double *sd, double *sa, double *u,
                    double *p, double *pr, double *res, long *dpnd, InfoPtr *info) {
  if (*dpnd) {
    *dpnd = RFCN_DPND(0, *sd, 0, 0, 0, 0);
    return;
  }
  VectorNd path_values = interpolator.getValues (*t);
  for (unsigned int i = 0; i < NoOfPos; i++) {
    res[i] = (path_values[i] - sd[i]);
  }
}
```

In LS-Barefoot/code/pathWalker2d.cc: The functions ffcn_… setup the right hand sides of the problem for each phase:

```
/* Right Hand Sides */
void ffcn_right_flat (double *t, double *xd, double *xa, double *u, double *p, double *rhs, double *rwh, long *iwh, InfoPtr *info) {
		walker.updateState (xd, u, p, CSRightFlat);
		walker.calcForwardDynamicsRhs (rhs);
}
void ffcn_right_halx (double *t, double *xd, double *xa, double *u, double *p, double *rhs, double *rwh, long *iwh, InfoPtr *info) {
		walker.updateState (xd, u, p, CSRightHalx);
		walker.calcForwardDynamicsRhs (rhs);
}
void ffcn_right_halx_left_heel (double *t, double *xd, double *xa, double *u, double *p, double *rhs, double *rwh, long *iwh, InfoPtr *info) {
		walker.updateState (xd, u, p, CSRightHalxLeftHeel);
		walker.calcForwardDynamicsRhs (rhs);
}
void ffcn_right_halx_left_flat (double *t, double *xd, double *xa, double *u, double *p, double *rhs, double *rwh, long *iwh, InfoPtr *info) {
		walker.updateState (xd, u, p, CSRightHalxLeftFlat);
		walker.calcForwardDynamicsRhs (rhs);
}
void ffcn_left_flat (double *t, double *xd, double *xa, double *u, double *p, double *rhs, double *rwh, long *iwh, InfoPtr *info) {
		walker.updateState (xd, u, p, CSLeftFlat);
		walker.calcForwardDynamicsRhs (rhs);
}
void ffcn_left_halx (double *t, double *xd, double *xa, double *u, double *p, double *rhs, double *rwh, long *iwh, InfoPtr *info) {
		walker.updateState (xd, u, p, CSLeftHalx);
		walker.calcForwardDynamicsRhs (rhs);
}
void ffcn_left_halx_right_heel (double *t, double *xd, double *xa, double *u, double *p, double *rhs, double *rwh, long *iwh, InfoPtr *info) {
		walker.updateState (xd, u, p, CSLeftHalxRightHeel);
		walker.calcForwardDynamicsRhs (rhs);
}
void ffcn_left_halx_right_flat (double *t, double *xd, double *xa, double *u, double *p, double *rhs, double *rwh, long *iwh, InfoPtr *info) {
		walker.updateState (xd, u, p, CSRightFlat);
		walker.calcForwardDynamicsRhs (rhs);
```

In LS-Barefoot/code/pathWalker2d.cc: Contact constraints for each phase are then defined with the functions rdfcn_…

The first phase, rdfcn_right_flat_s, consists of 10 constraints (rdfcn_right_flat_s_n = 10) of which the first 6 are equality constraints to be set to zero (rdfcn_right_flat_s_ne = 6). The vector res returns the residuals from the current constraint value.

```
static int rdfcn_right_flat_s_n = 10, rdfcn_right_flat_s_ne = 6;
void rdfcn_right_flat_s (double *ts, double *sd, double *sa, double *u, double *p, double *pr, double *res, long *dpnd, InfoPtr *info) {

  if (*dpnd) {
    *dpnd = RFCN_DPND(NULL, *sd, 0, *u, *p, 0);
    return;
  }

  walker.updateState (sd, u, p, CSRightFlat);

  Vector3d RightHeelPos = walker.getPointPosition (PointRightHeel);
  Vector3d RightHalxPos = walker.getPointPosition (PointRightHalx);
  Vector3d RightHeelVel = walker.getPointVelocity (PointRightHeel);
  Vector3d RightHalxVel = walker.getPointVelocity (PointRightHalx);
  Vector3d LeftHalxPos = walker.getPointPosition (PointLeftHalx);
  Vector3d RightHeelForce = walker.getPointForce (PointRightHeel);
  Vector3d RightHalxForce = walker.getPointForce (PointRightHalx);
  double limitingFriction = coeff_friction*(RightHeelForce[2]+RightHalxForce[2]);

  // Maintain Right Heel and Halx vertical positions on the ground
  res[0] = RightHeelPos[2];
  res[1] = RightHalxPos[2];

  // Maintain Right Heel velocities to zero
  res[2] = RightHeelVel[0];
  res[3] = RightHeelVel[2];

  // Maintain Right Halx anterior-posterior velocity to zero
  res[4] = RightHalxVel[2];

  // Maintain Left Halx vertical position on the ground
  res[5] = LeftHalxPos[2];

  // Maintain positive vertical force at Right Heel and Halx
  res[6] = RightHeelForce[2];
  res[7] = RightHalxForce[2];

  // Maintain anterior-posterior forces at Right Heel within friction cone
  res[8] = (limitingFriction-RightHeelForce[0]);
  res[9] = (limitingFriction+RightHeelForce[0]);
}
```

In LS-Barefoot/code/pathWalker2d.cc: Similarly constraints are defined for the other 7 phases. The subscripts _s, _i and _e denote start, interior and end point constraints for each phase.

rdfcn_right_flat_i
rdfcn_right_halx_s
rdfcn_right_halx_i
rdfcn_right_halx_left_heel_s
rdfcn_right_halx_left_heel_i
rdfcn_right_halx_left_flat_s
rdfcn_right_halx_left_flat_i
rdfcn_left_flat_s
rdfcn_left_flat_i
rdfcn_left_halx_s
rdfcn_left_halx_i
rdfcn_left_halx_right_heel_s
rdfcn_left_halx_right_heel_i
rdfcn_left_halx_right_flat_s
rdfcn_left_halx_right_flat_i

In LS-Barefoot/code/pathWalker2d.cc: Function def_model setups the OCP with each stage defined with the def_mstage function, for example,

```
// Right flat
def_mstage(
        0,
        nxd, nxa, nu,
        NULL, lfcn_reg,
        0, 0, 0, NULL, ffcn_right_flat, NULL,
        NULL, NULL
        );
```

In LS-Barefoot/code/pathWalker2d.cc: Function def_mpc setups the constraints for each stage, for example,

```
def_mpc (0, "s", npr, rdfcn_right_flat_s_n, rdfcn_right_flat_s_ne, rdfcn_right_flat_s, NULL);
def_mpc (0, "i", npr, rdfcn_right_flat_i_n, rdfcn_right_flat_i_ne, rdfcn_right_flat_i, NULL);
```

In LS-Barefoot/code/pathWalker2d.cc: Function def_lsq defines the least-squares objective function to be evaulated for each stage, for example,

```
def_lsq (0, "*", 0, nlsq, lsqfcn_trackMotion);
```

In LS-Barefoot/code/pathWalker2d_Model.cc: Function calcForwardDynamicsRhs() computes the joint accelerations (qddot), from the current joint angles (q), joint velocities (qvel), torques (tau_condensed), and active constraint set (constraintSets[activeConstraintSet]). Here, tau_condensed denotes the cumulative torque of the agonist-antagonist muscle torque generator. In our formulation we use the forward dynamics method proposed by Kokkevis and Metaxas “"Practical Physics for Articulated Characters”, Game Developers Conference, 2004, as implemented in the function ForwardDynamicsContactsKokkevis

```
void WalkerModel::calcForwardDynamicsRhs (double *res) {
        ForwardDynamicsContactsKokkevis (model, q, qdot, tau_condensed, constraintSets[activeConstraintSet], qddot);

        dynamicsComputed = true;
        unsigned int k = 0;
        for (unsigned int i = 0; i < PosNameLast; i++) {
          res[i] = qdot[i];
          res[i + PosNameLast] = qddot[i];
          k = i + PosNameLast + 1;
        }
        for (unsigned int j = 0; j < ControlNameLast; j++) {
          res[k] = adot[j];
          k = k + 1;
        }
}
```

In LS-Barefoot/code/pathWalker2d_Model.cc: Function setupTorqueMuscles() defines the agonist-antagonist muscle torque generators (MTGs) for each of the actuated joints.

```
void WalkerModel::setupTorqueMuscles (std::string datfilename, bool verbose) {
.
.
.
RightHipExtension_TorqueMuscle = Anderson2007TorqueMuscle (hip, ext, gender, age, subjectHeight, subjectMass, jointAngleOffset,
angleSign, torqueSign, "RhipExt");
.
.
.
RightHipExtension_TorqueMuscle.setMaximumActiveIsometricTorque(jointStrength[ParamRightHip_Ext]);
}
```

The source code for the MTGs appears in Dependencies/RBDL_pathWalker2d/addons/custom_forces. Compile the doxygen for RBDL-pathWalker2d for more information on the MTGs. Note that the MTG code has since been updated in later versions of RBDL and has been renamed to the ‘Millard2016TorqueMuscle’. However, the Anderson2007 curves can still be used within the updated code by using the Anderson2007 data set. See a recent release of RBDL for more details.

Code description MAPD-Barefoot

For this OCP, the decoupled constraint formulation and model setup are identical to the LS-Barefoot OCP.

In MAPD-Barefoot/code/pathWalker2d.cc: Additional periodic constraints (rcfcn_limit_cycle_s & rcfcn_limit_cycle_e) were added to the start of the first phase and end of the last phase, for example,

```
/* Coupled Constraints - This makes the states and controls at the end of the final stage identical to the start of the first phase */
static void rcfcn_limit_cycle_s (double *ts, double *sd, double *sa, double *u, double *p, double *pr, double *res, long *dpnd, InfoPtr *info) {
  if (*dpnd) {
    *dpnd = RFCN_DPND(0, *sd, *u, 0, 0, 0);
    return;
  }

  //Skip the x index.
  //StatePelvisPosX = 0,

  res[ 0] = sd[ StatePelvisPosZ              ];
  res[ 1] = sd[ StatePelvisRotY              ];
  res[ 2] = sd[ StateRightHipRotY            ];
  res[ 3] = sd[ StateRightKneeRotY           ];
  res[ 4] = sd[ StateRightAnkleRotY          ];
  res[ 5] = sd[ StateLeftHipRotY             ];
  res[ 6] = sd[ StateLeftKneeRotY            ];
  res[ 7] = sd[ StateLeftAnkleRotY           ];
  res[ 8] = sd[ StateTorsoRotY               ];
  res[ 9] = sd[ StatePelvisVelX              ];
  res[10] = sd[ StatePelvisVelZ              ];
  res[11] = sd[ StatePelvisRotVelY           ];
  res[12] = sd[ StateRightHipRotVelY         ];
  res[13] = sd[ StateRightKneeRotVelY        ];
  res[14] = sd[ StateRightAnkleRotVelY       ];
  res[15] = sd[ StateLeftHipRotVelY          ];
  res[16] = sd[ StateLeftKneeRotVelY         ];
  res[17] = sd[ StateLeftAnkleRotVelY        ];
  res[18] = sd[ StateTorsoRotVelY            ];
  res[19] = sd[ StateRightHipExtensionRotActY  ];
  res[20] = sd[ StateRightHipFlexionRotActY    ];
  res[21] = sd[ StateRightKneeExtensionRotActY ];
  res[22] = sd[ StateRightKneeFlexionRotActY   ];
  res[23] = sd[ StateRightAnkleExtensionRotActY];
  res[24] = sd[ StateRightAnkleFlexionRotActY  ];
  res[25] = sd[ StateLeftHipExtensionRotActY   ];
  res[26] = sd[ StateLeftHipFlexionRotActY     ];
  res[27] = sd[ StateLeftKneeExtensionRotActY  ];
  res[28] = sd[ StateLeftKneeFlexionRotActY    ];
  res[29] = sd[ StateLeftAnkleExtensionRotActY ];
  res[30] = sd[ StateLeftAnkleFlexionRotActY   ];
  res[31] = sd[ StateTorsoExtensionRotActY     ];
  res[32] = sd[ StateTorsoFlexionRotActY       ];

  res[33] = u[ControlRightHipExtensionRotY  ];
  res[34] = u[ControlRightHipFlexionRotY    ];
  res[35] = u[ControlRightKneeExtensionRotY ];
  res[36] = u[ControlRightKneeFlexionRotY   ];
  res[37] = u[ControlRightAnkleExtensionRotY];
  res[38] = u[ControlRightAnkleFlexionRotY  ];
  res[39] = u[ControlLeftHipExtensionRotY   ];
  res[40] = u[ControlLeftHipFlexionRotY     ];
  res[41] = u[ControlLeftKneeExtensionRotY  ];
  res[42] = u[ControlLeftKneeFlexionRotY    ];
  res[43] = u[ControlLeftAnkleExtensionRotY ];
  res[44] = u[ControlLeftAnkleFlexionRotY   ];
  res[45] = u[ControlTorsoExtensionRotY     ];
  res[46] = u[ControlTorsoFlexionRotY       ];
}}
```

In MAPD-Barefoot/code/pathWalker2d.cc: The objective function in this OCP minimized squared activation per distance walked. Note that here we integrate the activation over all phases with the ffcn_cost function before dividing by the total distance walked sd[StatePelvisPosX] in the function mfcn,

```
void mfcn (double *t, double *sd, double *sa, double *p, double *pr, double *mval, long *dpnd, InfoPtr *info) {

  if (*dpnd) { *dpnd = MFCN_DPND(*t, *sd, 0, 0, 0); return; }

  *mval = 0.;
  *mval = sd[MayerStateActivationSquared] / (1e-6 + fabs(sd[StatePelvisPosX]));
}

void ffcn_cost (double *t, double *xd, double *xa, double *u, double *p, double *rhs, double *rwh, long *iwh, InfoPtr *info){
    rhs[MayerStateActivationSquared] = 0;
    double val = 0;
    for(int i = StateRightHipExtensionRotActY; i <= StateTorsoFlexionRotActY; ++i){
      val += xd[i]*xd[i];
    }
    rhs[MayerStateActivationSquared] = val;
}
```

Code description MAPD-Orthosis

The decoupled and periodic constraints, model setup, as well as the objective function formulation are identical to the MAPD-Barefoot OCP.

In MAPD-Orthosis/code/pathWalker2d.cc: Four additional free parameters of the problem are introduced to identify the optimal stiffness of the ankle-foot orthosis (in code/pathWalker2d_Enums.h). Note that only the last 4 parameters are allowed to vary in the OCP.

```
enum ParamName {
		ParamRightHip_Ext = 0,
		ParamRightHip_Flex,
		ParamRightKnee_Ext,
		ParamRightKnee_Flex,
		ParamRightAnkle_Ext,
		ParamRightAnkle_Flex,
		ParamLeftHip_Ext,
		ParamLeftHip_Flex,
		ParamLeftKnee_Ext,
		ParamLeftKnee_Flex,
		ParamLeftAnkle_Ext,
		ParamLeftAnkle_Flex,
		ParamTorso_Ext,
		ParamTorso_Flex,
		ParamRightOrthosisStiffnessInDorsiFlexion,
		ParamRightOrthosisStiffnessInPlantarFlexion,
		ParamLeftOrthosisStiffnessInDorsiFlexion,
		ParamLeftOrthosisStiffnessInPlantarFlexion,
		ParamNameLast
};
```

Code description MAPD-WS-Orthosis

This problem is identical to MAPD-Orthosis.

In MAPD-WS-Orthosis/code/pathWalker2d.cc: An additional term in the objective function to favor higher walking speed:

```
void mfcn (double *t, double *sd, double *sa, double *p, double *pr, double *mval, long *dpnd, InfoPtr *info) {

 if (*dpnd) { *dpnd = MFCN_DPND(*t, *sd, 0, *p, 0); return; }

 *mval = 0.;
 *mval = sd[MayerStateActivationSquared] / (1e-6 + fabs(sd[StatePelvisPosX]));
 *mval += (-sd[StatePelvisPosX] / *t ) * 2.0;
}

void ffcn_cost(double *t, double *xd, double *xa, double *u, double *p, double *rhs, double *rwh, long *iwh, InfoPtr *info){
   rhs[MayerStateActivationSquared] = 0;
   double val = 0;
   for(int i = StateRightHipExtensionRotActY; i <= StateTorsoFlexionRotActY; ++i){
     val += xd[i]*xd[i];
   }
   rhs[MayerStateActivationSquared] = val;
}
```
