# Supplementary material for: Optimal Control Based Stiffness Identification of an Ankle-Foot Orthosis Using a Predictive Walking Model
Source: Front Comput Neurosci. 2017 Apr 13;11:23. doi: 10.3389/fncom.2017.00023 (PMC5390028; doi:10.3389/fncom.2017.00023)
Supplement: Supplementary file 1 [file DataSheet1.ZIP › PathWalker/README.pdf]

# PathWalker Distribution
# Description of data and software layout

*Supplementary material for the study "Optimal control based stiffness identification of an ankle-foot orthosis using a predictive walking model", by M. Sreenivasa, M. Millard, M. Felis, K. Mombaur & S.I. Wolf*
*Contact: M. Sreenivasa <manish.sreenivasa@iwr.uni-heidelberg.de>,*
*Heidelberg University, Germany*

This document lists the directories, subdirectories and main content pertaining to the PathWalker2d data and software. Detailed descriptions of the constraint formulations and OCP software code are available in the document “PathWalker_CodeDescription.pdf”. Please note that the c++ code is not meant to be compiled as the dependency Muscod is not available as open-source code.

**1. Data** – contains experimental recordings of the patient walking barefeet and with bilateral ankle-foot orthoses. This data can be viewed with the open-source visualization tool Mokka, distributed with the BTK software: https://github.com/Biomechanical-ToolKit/BTKCore

**2. Models** – contains the whole body models of the patient with and without orthoses in Lua format. The models may be visualized using the open-source softwares Puppeteer and Meshup: https://bitbucket.org/MartinFelis/meshup

**3. InvKinDyn** – contains code to compute inverse dynamics (results included) and plot the results.

- a. code – contains c++ code to compute the inverse dynamics. Install RBDL and Puppeteer using the version distributed in the dependencies folder, to run the programs run_ik and run_id.
- b. results – precomputed inverse kinematics and inverse dynamics results
- c. plots – matlab/octave code to plot the results

**4. LS-Barefoot** – contains code and results from the OCP minimizing differences from recorded kinematics during barefoot gait. Includes plotting functions. See “PathWalker_CodeDescription.pdf” for details about constraint formulation and model setup.

- a. code
- b. results

**5. MAPD-Barefoot** – contains code and results from the OCP minimizing activations squared per distance walked for barefoot gait. Includes plotting functions. See “PathWalker_CodeDescription.pdf” for details about constraint formulation and model setup.

- a. code
- b. results

**6. MAPD-Orthosis** - contains code and results from the OCP minimizing activations squared per distance walked for orthosis gait. Includes plotting functions. See “PathWalker_CodeDescription.pdf” for details about constraint formulation and model setup.

- a. code
- b. results

**7. MAPD-WS-Orthosis** - contains code and results from the OCP minimizing activations squared per distance walked for orthosis gait, favoring higher walking speed. Includes plotting functions. See “PathWalker_CodeDescription.pdf” for details about constraint formulation and model setup.

a. code
b. results

**8. Dependencies** – versions of the open-source softwares RBDL and Puppeteer used to compute the results.
